# Antiviral Efficacy of Selected Natural Phytochemicals against SARS-CoV-2 Spike Glycoprotein Using Structure-Based Drug Designing

**DOI:** 10.3390/molecules27082401

**Published:** 2022-04-08

**Authors:** Bandar Hamad Aloufi, Mejdi Snoussi, Abdel Moneim E. Sulieman

**Affiliations:** 1 Department of Biology, College of Science, University of Hail, P.O. Box 2440, Ha’il 2440, Saudi Arabia; Bandaraloufi@yahoo.com (B.H.A.); abuelhadi@hotmail.com (A.M.E.S.); 2 Laboratory of Genetics, Biodiversity and Valorisation of Bioressources, High Institute of Biotechnology, University of Monastir, Monastir 5000, Tunisia

**Keywords:** SARS-CoV-2, phytochemicals, molecular docking, drug targets, molecular dynamic simulation

## Abstract

SARS-CoV-2 is a highly virulent coronavirus that first surfaced in late 2019 and has since created a pandemic of the acute respiratory sickness known as “coronavirus disease 2019” (COVID-19), posing a threat to human health and public safety. S-RBD is a coronaviral protein that is essential for a coronavirus (CoV) to bind and penetrate into host cells. As a result, it has become a popular pharmacological target. The goal of this study was to find potential candidates for anti-coronavirus disease 2019 (COVID-19) drugs by targeting severe acute respiratory syndrome coronavirus 2 (SARS-CoV-2) S-RBD with novel bioactive compounds and molecular interaction studies of 15,000 phytochemicals belonging to different flavonoid subgroups. A spike protein crystal structure attached to the ACE2 structure was obtained from the PDB database. A library of 15,000 phytochemicals was made by collecting compounds from different databases, such as the Zinc-database, PubChem-database, and MPD3-database. This library was docked against a receptor binding domain of a spike glycoprotein through the Molecular Operating Environment (MOE). The top drug candidates Phylloflavan, Milk thistle, Ilexin B and Isosilybin B, after virtual screening, were selected on the basis of the least binding score. Phylloflavan ranked as the top compound because of its least binding affinity score of −14.09 kcal/mol. In silico studies showed that all those compounds showed good activity and could be used as an immunological response with no bioavailability issues. Absorption, distribution, metabolism, excretion and a toxicological analysis were conducted through SwissADME. Stability and effectiveness of the docked complexes were elucidated by performing the 100 ns molecular dynamic simulation through the Desmond package.

## 1. Introduction

Coronavirus (CoV) is a virus encircled by a single-stranded, positive-sense RNA genome that is believed to produce human respiratory infections. The world has been grasped in a pandemic by the attack of the acute respiratory disease coronavirus (SARS-CoV-2) that was established in 2019. The world’s culture, economy, healthcare and infrastructure of its population has been challenged by this pandemic [1,2]. In December 2019, an unusual novel and viral pneumonia originated in Wuhan, China. It has been recognized as a zoonotic disease similar to SARS-CoV and MERS-CoV and, hence, given the name nCoV-2019. In January 2020, a patient affected by the coronavirus was observed using the throat swab test by the Chinese center for disease control and prevention and, consequently, it was identified as nCoV-2019 by the World Health Organization [3,4]. This epidemic illness caused by SARS-CoV-2 was announced as coronavirus disease (COVID-19) [4]. Once a person has become infected with corona virus, it takes 2–14 days for the appearance of symptoms [5]. A period of 5–6 days is roughly considered as the average incubation period [6]. As per the statement of the World Health Organization, symptoms of COVID-19 are minor and appear gradually [7]. The Lancet exclaimed, when admission in the hospital is required, this usually takes place after 7 days [8].

The chemical characterization and analysis of the composition of plant materials provides the scientific basis necessary for the invention and formation of new drugs of natural sources. Phytochemicals are basically natural compounds present in vegetables, fruits, medicinal plants, aromatic plants, leaves, flowers and roots [9]. Depending on their function, in plant metabolism these are characterized as primary metabolites (carbohydrates, proteins and lipids) and secondary metabolites (polyphenols, steroids, alkaloids, carotenoids, etc.) [10,11]. This research summarizes the available evidence for the effect of photochemistry on multiple diseases. A high usage of fresh fruits and vegetables is probably associated with a low risk of mortality attributed to the prevention of COVID-19 [12,13]. Phytochemicals are the parts of plants that protect themselves from environmental threats, including water changes and microbes, and give their color, flavor, scent and texture [14]. Alkaloids, flavonoids, phenols and tannins are natural phytochemicals found in plants that have been studied as potential antioxidants [15]. Many acute and chronic diseases, such as diabetes, asthma, Parkinson’s, atherosclerosis, cancer, cataracts, neurological disorder, liver injury and human ageing, are linked to free radicals and other reactive oxygen species [16,17]. Antioxidants, on the other hand, are helpful molecules that help to delay and reduce the action of such drugs by preventing oxidative damage to the target locations [18].

After an incubation period of approximately 5–7 days, symptoms of coronavirus appear. Once the person interacts with coronavirus, it takes 6–41 days from the warning signs to death [19]. The period length depends on the person’s age, as well as the status of the immune response of the person. Persons above 70 years have shorter periods compared with persons under 70 years of age [20]. Fever was found in 86–90% of the patients. Other symptoms include fatigue, cough, mucus production, headache, diarrhea, hemoptysis, lymphopenia and dyspnea [21]. There are similarities that are vital to note between COVID-19 and previous beta coronaviruses, such as fever and dry cough, as well as dyspnea [22]. However, some clinically distinctive features are observed in COVID-19 that consist of the targeting of the lower airway, as made obvious by upper respiratory tract signs such as sneezing and rhinorrhea, as well as sore throat [23].

During research, clinical data from other types of coronaviruses proposed larger tissue intrusiveness, as well as an apparent neurotropism that can lead to more complicated clinical circumstances [24]. It was revealed that coronaviruses, particularly beta coronaviruses to which SARS-CoV-2 belongs, do not restrict their existence to only the respiratory tract, but also spread to the central nervous system. On 2nd March, a Saudi citizen traveling from Iran via Bahrain screened positive for COVID-19, and the Health ministry quickly isolated and declared the occurrence as the first in the Kingdom of Saudi Arabia [25]. Since the outbreak began, the country has seen 750,356 illnesses and 9039 coronavirus-related deaths [26]. It has been demonstrated that SARS-CoV and MERS-CoV, in addition to the coronavirus, are responsible for porcine hemagglutinating encephalomyelitis (HEV 67N) [27,28]. Bioinformatics tools, programs, and databases are heavily used in computer assisted drug design (CADD) techniques. As a result, computer-assisted drug design science and computational biology have a lot in common [29].

## 2. Results

### 2.1. Structure Retrieval of Spike Protein

**Figure 1:**
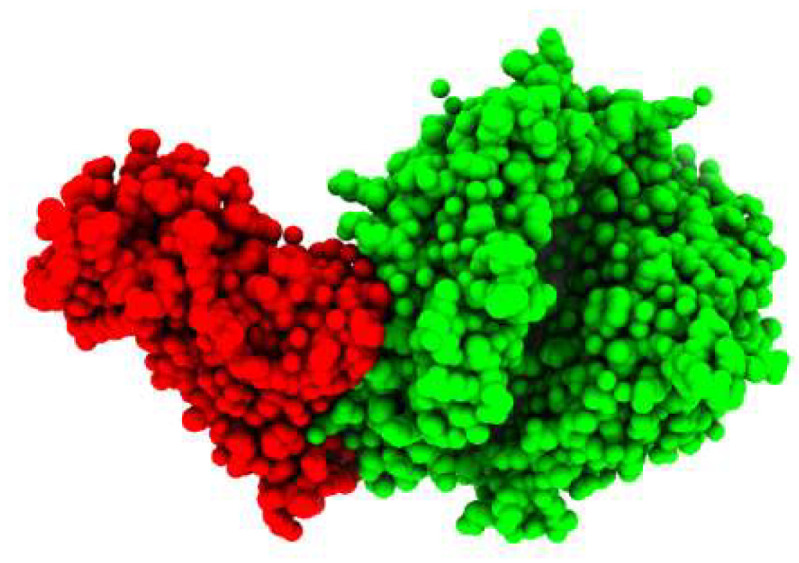
3D-Visulization of the spike glycoprotein receptor with human ACE2 enzyme.

The crystal structure of the COVID-19 spike receptor-binding domain (RBD) bound to the ACE2 (Figure 1) was selected as the target (receptor) from PDB ID (6LZG). In order to gain insight into the relationship between the H-ACE2 and the SARS-CoV-2 spike receptor-binding domain, we first studied the spike receptor-binding domain and the H-ACE2 complex interfaces. Some potent spike receptor-binding domain residues significantly bonded to the ACE2, leading to the stability of complexity.

### 2.2. Database Screening and Docking Study

The spike protein was docked against a ligand molecular library of 15,000 phytochemicals. Docking was a reliable procedure which showed the relationship between phytochemicals and the SARS-CoV-2 spike protein receptor-binding domain. MOE software was used to perform molecular docking in order to find compounds with the best binding residue-to-receptor interaction. The RMSD value, the residue binding with ligands and the docking score were used to evaluate compounds. The top six ranked docking poses were picked from 15,000 docked molecules. Compounds with a low score, such as rmsd3, and residues with the most interactions were chosen. The lowest binding energy of these selected phytochemicals ranged from −14.09 to −12.19 (kcal /mol). In order to obtain a precise sense of the receptor-ligand interaction with the highest docked complexes, the minimum binding energy and scoring function of each docked complex were shown in Table 1.

**Figure 2:**
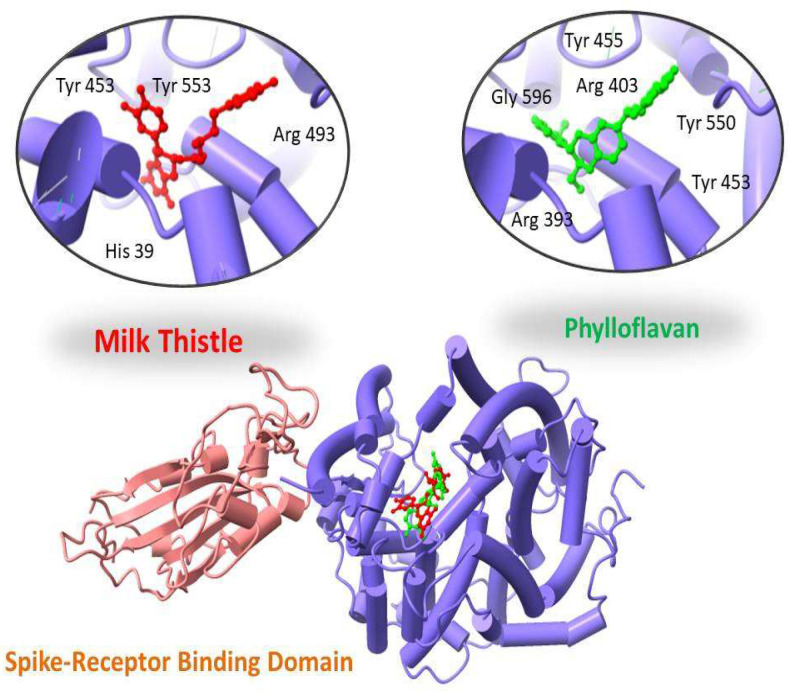
Three-dimensional visualization of top two docked complexes (Phylloflavan and Milk thistle) with spike glycoprotein forming hydrogen bonds with the side chains.

Two-dimensional maps of these interactions were analyzed using the MOE LigX tool. ChimeraX was used to display docked complexes. The LigX interaction diagram showed that Phylloflavan and Milk thistle were found to bind with the spike receptor binding domain protein with a score of −14.09 kcal/mol and −13.10 kcal/mol, forming hydrogen bonds and other interactions with the side chains of Tyr 455, Tyr 550, Arg 393, Gly 596, Tyr 453 and Arg 403 (Phylloflavan), and Tyr 453, Tyr 553, Arg 493 and His 39 (Milk thistle), as shown in Figure 2.

**Figure 3:**
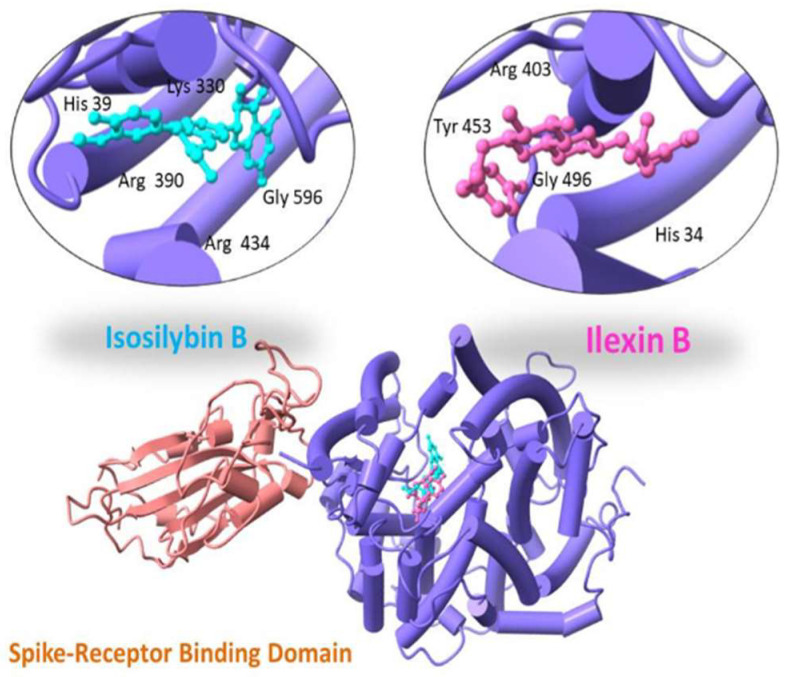
Three-dimensional visualization of top two docked complexes (Ilexin B and Isosilybin B) with spike glycoprotein forming hydrogen bonds with the side chains.

With a binding score of −13.04 kcal/mol, Ilexin B showed hydrogen bonding with Tyr 453, Gly 496, His 34 and Arg 403. Isosilybin B showed a binding score of −12.19 kcal/mol with interacting residues Arg 390, Arg 434, Lys 330, His 39 and Gly 596, as shown in Figure 3.

### 2.3. Drug-Likeness/ADMET Profiling

To analyze the drug-likeness qualities of the top compounds, a drug scanning was performed using the Molinspiration service. Lipinski’s Rule of Five has become a norm; this rule depicts important drug features such as pharmacokinetics, interactions and metabolism in the human body, as well as their excretion. The selected compounds had no violations of Lipinski’s five rules and had significant drug-like features, such as molecular weight (Table 2).

ADME and AdmetSAR were used to assess a number of pharmacokinetic variables. The ADME and toxicity of the top therapeutic candidate medications can be estimated using pharmacokinetic characteristics. Table 3 shows the ADMET properties of derived phytochemicals for both targets. Due to poor pharmacokinetic qualities and toxicity, many medicines do not exploit this mechanism in their development. Early drug discovery relies on high-performance and quick ADMET profiling investigations to identify active lead compounds. SwissADME was also utilized to test the ADMET profiles of prospective compounds to validate the drug likeliness (Table 3).

### 2.4. Biochemical Classification of Idetified Compounds

All the compounds’ classifications and their use against diseases were previously explained in Table 4. All structures and chemical properties, including taxonomy and its biological classification, were explained for a better understanding of the efficacy of our predicted drug candidates.

### 2.5. Energy Calculations

The binding free energy (deltaG bind) of inhibitory drugs with a strong potential to inhibit the activity of the SARS-CoV-2 spike protein was calculated using MMGBSA. To perform the MMGBSA energy estimates, docking complexes with high energy function scores were obtained. By minimizing protein-ligand complexes, salvation energy and surface area energy, total free binding energy was determined. The results revealed that the compound name with ID had a high level of stability, with a total binding free energy of −30.35 kcal/mol for the Phylloflavan compound, −28.90 kcal/mol for Milk thistle, −31.83 kcal/mol for Ilexin B and −34.97 kcal/mol for Isosilybin B, as shown in Table 5.

### 2.6. MD Simulation

Docking analysis was used to determine the best position for the ligand to bind strongly to the receptor. The interaction patterns of the top complex with the target spike-receptor binding protein were determined using the MD simulation. After the MD simulation, the RMSD, RMSF, SASA and SSE distribution analyses were performed.

#### 2.6.1. Root Mean Square Deviation (RMSD)

**Figure 4:**
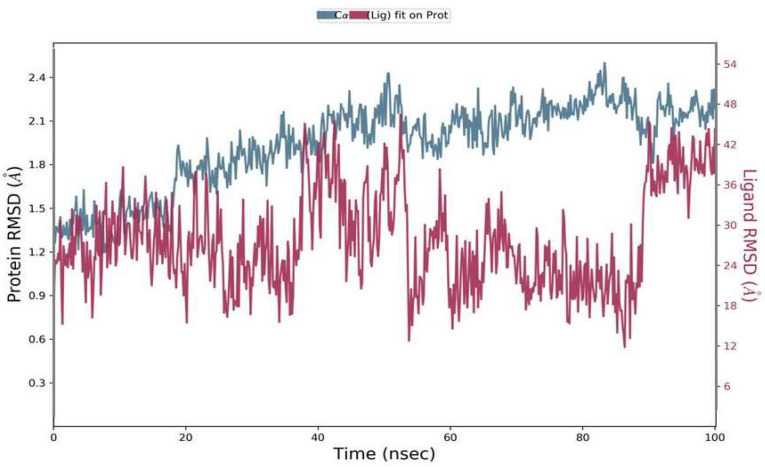
Residual flexibility was calculated using the root mean square deviation (RMSD) during a 100-ns time period.

The RMSD was calculated during 100 ns simulations for the top complex. The RMSD was used as a function of time for the best ligand (Phylloflavan) with the spike-receptor binding domain protein. The RMSD results revealed that the MD simulation was equilibrated between 1 Å and 1.8 Å (Figure 4). Although it showed stability up to 50 ns, after that it showed minor deviations up to 90 ns; following that, it showed stability again. The RMSD results signified that the spike-receptor binding domain did not undergo large conformational changes. The RMSD plot of ligands in the right *y*-axis direction suggested that all these ligands were stable during simulation, with respect to the protein binding pocket.

#### 2.6.2. Root Mean Square Fluctuations

**Figure 5:**
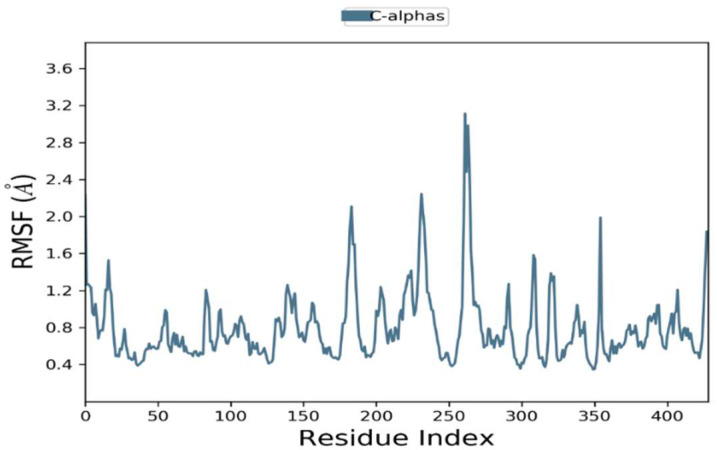
Residual flexibility of root mean square fluctuations (RMSFs) were demonstrated during a 100 ns time period.

The RMSFs were calculated to examine residential stability and flexibility over 100 ns. Trajectory conformation variability can be modified to compute the RMSFs for individual atoms. Phylloflavan had an average RMSF of 1.2 for overall positions. At the C-terminal and N-terminal, there were significant differences in all trajectories. The complexes’ total residual fluctuations are depicted in Figure 5.

#### 2.6.3. Solvent Accessible Surface Area (SASA)

**Figure 6:**
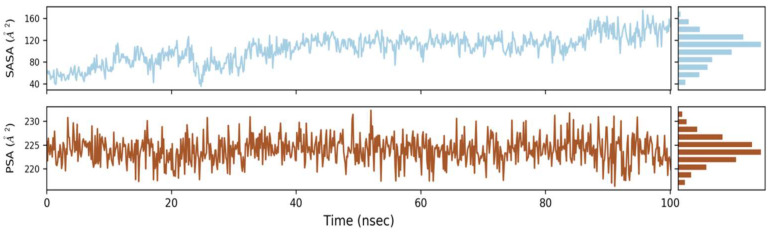
Graphs of solvent accessible surface area (SASA) revealed the protein’s stability and folding.

SASA stands for solvent accessible surface area, and it is a new approach to keep proteins stable and folded. Figure 6 shows the computed SASA values for wild types and mutants. The average SASA values for Phylloflavan were determined to be 40, 80, 120 and 160, respectively, indicating that the available area of all the systems did not vary much over the simulation procedure.

#### 2.6.4. Water Bridges, Ionic Interactions and Hydrogen Bonding Graphs

**Figure 7:**
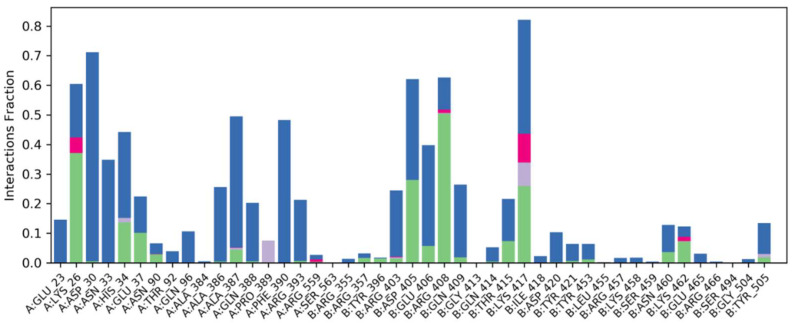
After MD simulation, strong water bridges, ionic contacts and generated hydrogen bonding graphs can be seen in docked Phylloflavan in complex with spike glycoprotein.

Understanding the binding mechanism of both complexes in the binding pocket of the spike-receptor binding domain necessitates atomic-level knowledge. Hydrogen bonding, ionic interaction, hydrophobic contacts and salt bridges are all essential intermolecular interacting forces in binding mode analysis. Over 100 ns simulation studies, these intermolecular forces of interactions were predicted (Figure 7).

## 3. Discussion

The best method to grasp the host-specific, infectious and pathogenic nature of COVID-19 is to fully comprehend the process for identifying viral receptors, which is critical for the creation of remedial treatments, medicines and antiviral cures [30]. There is mounting evidence that ACE2 gene polymorphism affects the association between ACE2 and the SARS-CoV-2 spike protein, influencing the viral entrance into the host organism and contributing to COVID-19 lungs and systemic harm [31]. Until now, none of the medications or treatments were proven to be effective against SARS-CoV-2, and the use and development of new medications is expensive and time-consuming due to the numerous testing stages required [32,33,34]. Worse, the WHO predicts that COVID-19 will become endemic, signaling to the scientific community that treatment development for it has grown more critical. In this sense, docking methods are technically developed as a highly specific inhibitor for viral proteins, as well as an antiviral medication research and development process [35]. The worldwide risk posed by COVID-19 has prompted a rapid search for a therapeutic agent and the use of biological technologies, such as docking studies, as well as bioinformatics tools, to quickly assess the efficiency of other available medications against severe acute respiratory 2 infections [36].

A spike protein is a type I glycoprotein that protrudes from the surface of the virus and is the first component to make contact with the host cell. The establishment of COVID-19 treatment techniques is critical since the spike protein is the virus’ key component for binding receptors to the host surface. An S-protein in the SARS-CoV-2 envelope is responsible for the interaction between host cells and the ACE2 receiver via the RBD unit [37]. This is the first and most important step in a SARS-CoV-2 infection and the interaction with the host cells. In the fight against SARS-CoV-2 inflammation, an antiviral medication that targets S-RBD–ACE2, which can transfer viruses into a host cell, gives a quick result [38,39].

Scientists have lately used computational capabilities to estimate the likelihood of joining numerous molecules before estimating and developing them in the lab. Molecular docking is used to find the binding pattern of small molecules against their target. As a result, molecular docking appears to be a valuable strategy for creating and screening novel chemicals to combat devastating diseases. The molecular interactions of 15,000 phytochemicals with different flavonoid subgroups and S-RBD–ACE2 were investigated. Structure optimization of ligand molecules and energy reduction in S-RBD–ACE2 were performed prior to docking analysis.

## 4. Materials and Methods

### 4.1. Data Collection and Ligand Database

For molecular docking, the 2D conformation of 15,000 phytochemicals were retrieved from six different databases, i.e., from MAPS database [40], PubChem [41], Zinc database [42], MPD3 [43], ChEMBL [44] and NPACT [45]. Each molecule of ligand was saved into the MOE database in mol form after minimization of energy. All of these were conducted through MOE. Drug designing, protein structure analysis, data processing and docking; all these steps were conducted through MOE.

### 4.2. Receptor Preparation and Analysis of Target Active Binding Sites

MOE performed molecular docking of the terpenoids dataset [46]. The l structure of the spike protein coupled to the ACE2 protein structure was retrieved from the PDB for docking purposes (PDB ID 6LZG) [47]. The MOE software’s site finder function located the target protein’s active sites. The ligand coordinates defined the active site in the original target protein site. The binding pocket containing the catalytic triad was selected.

### 4.3. Molecular Docking

Molecular docking is a computational procedure that is used to check the interaction between molecule and protein at an atomic level [48]. Various types of computational approaches are utilized to predict the affinity between the target and the active site [49]. Rigid ligand docking, semi-flexible ligand docking, and flexi docking are the three types. A particular scoring location confirms the ligand’s approval in sub-atomic docking. There are specific scoring positions: in molecular docking, either a systematic or a ligand arch is used to evaluate a conformational search [50]. In molecular docking, binding energetics were tested using a force-field based scoring method, an empirical scoring function and a knowledge-based scoring function [51].

### 4.4. Analysis of Ligand Receptor Interaction

MOE’s LigX tool was used to analyze the receptor-ligand interaction on 2D plots, in order to obtain a high-quality image of the top-docked complexes’ receptor-ligand interaction. It showed a 2D graph of electrostatic contacts, hydrogen bonding, hydrophobic interactions and Van der Waals forces that were taken into account when determining the affinity of the drug-like molecule within the actively docked pockets. MOE is a program that creates three-dimensional pictures of protein-inhibitor complexes [52].

### 4.5. Physiochemical Property Profile and Toxicity Prediction

All the selected molecules were analyzed through the Molinspiration server for Lipinski’s Rule of Five. This rule explains different drugs’ properties, such as absorption, metabolism and drug secretion in the human body. Although the different drugs are evaluated on the basis of this rule, it also includes different values, such as MW, hydrogen HBA, HBD and log *p*-value. These are the standard of these values (A log *p* < 5, fewer than 10 H-bond acceptors, fewer than 5 H-bond donors and a molecular weight of fewer than 500 Daltons) [53]. Swiss-ADME software was used to predict the pharmacokinetic properties (absorption, metabolism, distribution, excretion and toxicity) [54].

### 4.6. MM-GBSA Binding Free Energy Calculations

Docking complexes were further validated by calculating the binding free energy (Prime/MM-GBSA) using the Schrodinger Suit Release 2020 [55]. The best poses of the inhibitory compounds related to the SARS-CoV-2 spike protein were chosen to obtain binding-free energies. The local optimization feature was used to minimize the docked complexes in Prime. Prime MM-GBSA was used to calculate the binding free energies based on the combination of the OPL-SAA force field, EMM (Molecular Mechanics Energies), the GSGB (solvation model for the polar solvation), the nonpolar solvation term (GNP) made up of solvent accessible surface area (SASA) and Van der Waals interactions. The binding free energy calculations were conducted based on the following equation: ∆Gbind = GComplex − Gprotein + Gligand.

### 4.7. Molecular Dynamic Simulation

Stability and effectiveness of the docked complexes were elucidated by performing the 100 ns molecular dynamic simulation [56]. The simulation was carried out using the Desmond package, which included a solvent system and a force-field called Optimized Potentials for Liquid Simulations 3 (OPLS3) [57]. Water molecules were used to solvate the molecular system. The system was electrically neutralized using Na/Cl ions. Before the MD simulation, the system used a heating process to reduce its energy use. Complexes were also reduced utilizing a steepest descent steps-based approach as part of the reduction method. Furthermore, after 1,000 sharpest descent steps, the system was brought into balance. Finally, the simulation was performed for 100 ns time at 300 K temperature and at 1 atm pressure using the NPT-ensembles.

## 5. Conclusions

The current study used an in silico approach encompassing many phases to recognize and identify active pharmacological targets for intimate drug creation against SARS-CoV-2. The goal of this research was to find druggable targets. Disease searching, disease-related genes, docking interaction and evaluation of docked complexes for medication potency were some of the bioinformatics and computational techniques utilized to follow the workflow. To select the compounds having the best residue interaction with the target protein, molecular docking was used. After examining the top 100 docked compounds, six molecules were chosen as the best molecules based on their docking score and drug evaluation. Phylloflavan, Milk thistle, Ilexin B and Isosilybin B were found as promising phytochemicals in the current investigation, with a good binding capability to SARS-CoV-2 S-RBD, and all have drug-like qualities. The findings of this study can be used to create and develop new drugs that have improved inhibitory activity on the spike glycoprotein. Although, for findings validation, an in vitro and in vivo experimental study is recommended.

## Figures and Tables

**Table 1:** Indicating the binding affinity along with interacting residues.

IDs (PubChem)	Phytochemicals	Binding Affinity (kcal/mol)	RMSD Value	Hydrogen Bonds and Other Interacting Residues
**457885**	Phylloflavan	−14.09	0.59	Tyr 455; Tyr 550; Arg 393; Gly 596; Tyr 453; Arg 403
**1548994**	Milk thistle	−13.10	1.01	Tyr 453; Tyr 553; Arg 493; His 39
**102394711**	Ilexin B	−13.04	1.53	Tyr 453; Gly 496; His 34; Arg 403
**10885340**	Isosilybin B	−12.19	0.92	Arg 390; Arg 434; Lys 330; His 39 Gly 596

**Table 2:** According to the Lipinski rule, these compounds have a strong probability of becoming drugs.

Sr No	Compounds Name	Log P	M Weight	HBD	HBA
1	Phylloflavan	2.53	498.48	10	4
2	Milk thistle	−1.25	481.43	10	4
3	Ilexin B	−1.82	464	10	2
4	Isosilybin B	−1.25	481.43	10	4

**Table 3:** Potential compounds’ ADMET profiling of top drug candidates.

Compounds	Phylloflavan	Milk Thistle	Ilexin B	Isosilybin B
**Absorption**				
**Blood-Brain Barrier**	No	No	No	No
**Distribution**				
**Gastro-Intestinal-Absorption**	Low	Low	Low	Low
**P-glycoprotein-substrate**	No	No	Yes	No
**CYP450-1A2-Inhibitor**	No	No	No	No
**Metabolisum**				
**CYP450-2C9-Inhibitor**	No	No	No	No
**CYP450-2D6-Inhibitor**	No	No	No	No
**CYP450-2C19-Inhibitor**	No	No	Yes	No
**CYP450-3A4 Inhibitor**	No	No	No	No
**Toxicity**				
**Cytotoxicity**	Non-toxic	Non-toxic	Non-toxic	Non-toxic
**Immunogenicity**	Non-toxic	Non-toxic	Non-toxic	Non-toxic
**Mutagenicity**	Non-toxic	Non-toxic	Non-toxic	Non-toxic

**Table 4:** Biochemical classification of reported drug candidates.

Compounds	Taxonomy	Classification	Diseases
Phylloflavan	Phyllocladus trichomanoides Phyllocladus alpinus	Polyketides Flavonoids Flavans, Flavanols and Leucoanthocyanidins	Antileishmanial activity and modulatory effects on nitric oxide and tumor necrosis human immunodeficiency virus type 1 integrase
Milk thistle	Anastatica hierochuntica Silybum marianum Aspergillus iizukae	Flavonoids silibinin dehydrosilibinin silychristin silydianin	Liver disorders and gallbladder problems.hepatitis, cirrhosis, jaundice, diabetes, indigestion
Ilexin B	Panax notoginseng	Glucosides Carbohydrates	Inflammatory bowel disease, arthritis, ischemia, atherosclerosis, Alzheimer disease and trauma, as well as hyperlipidemia, diabetes
Isosilybin B	Anastatica hierochuntica Silybum marianum	Hydrocarbons, Aromatic Hydrocarbons, Cyclic Benzene Derivatives Flavonolignans	Antiprostate cancer activity via inhibiting proliferation and inducing G1 phase arrestand apoptosia.

**Table 5:** MM-PBSA energy calculations for all complexes.

Energy Parameters	VDWAALS (kcal mol^−1^)	Delta G Gas (kcal mol^−1^)	Delta g Solv (kcal mol^−1^)	Delta Total (kcal mol^−1^)
Phylloflavan/S-RBD	−29.50	−34.56	8.21	−30.35
Milk thistle/S-RBD	−30.61	−31.87	9.86	−28.90
Ilexin B/S-RBD	−31.70	−29.54	10.23	−31.83
Isosilybin B/S-RBD	−28.74	−32.33	11.23	−34.97

## Data Availability

The data generated and analyzed during this study are included in this article.
